# Error disclosure: what residents say and what patients find effective

**DOI:** 10.3389/frhs.2025.1577092

**Published:** 2025-06-20

**Authors:** Emily Grossniklaus, Angelo D'Addario, Ann King, Thomas H. Gallagher, Kathleen Mazor, Andrew A. White

**Affiliations:** ^1^ Department of Medicine, VA Puget Sound Healthcare System and University of Washington School of Medicine, Seattle, WA, United States; ^2^ Office of Research Strategy, National Board of Medical Examiners, Philadelphia, PA, United States; ^3^ Department of Medicine and Department of Bioethics, University of Washington School of Medicine, Seattle, WA, United States; ^4^ Department of Medicine, UMass Chan Medical School, Worcester, MA, United States; ^5^ Department of Medicine, University of Washington School of Medicine, Seattle, WA, United States

**Keywords:** error disclosure, communication training, graduate medical education, communication skills assessment, crowdsourcing

## Abstract

**Background:**

Medical error disclosure to patients is a critical skill that is often not taught effectively in medical training. The Video-based Communication Assessment (VCA) software enables trainees to receive feedback on their error disclosure communication skills. The VCA method also allows examination of the specific types of error disclosure responses that patients value most.

**Objective:**

The primary aim of this study was to describe the language medical residents use to disclose a hypothetical harmful medical error, and to determine the language associated with higher ratings by crowdsourced laypeople. A secondary aim of this study was to examine the alignment between error disclosure content recommended by experts and the communication behaviors that contribute to higher layperson ratings of disclosure.

**Methods:**

102 resident physician responses to a case depicting a delayed diagnosis of breast cancer and their crowdsourced ratings were analyzed using thematic content analysis. We assessed the presence of specific themes in response to three sequential video prompts within a clinical case. Linear regressions were then performed for each prompt's response to examine the extent to which each theme predicted overall communication scores from layperson raters.

**Results:**

Nearly all (*N* = 92, 90.2%) residents provided responses which included either a general apology or a specific apology in at least one of the three prompt's responses, and nearly all (*N* = 98, 96.1%) residents provided at least one response expressing a component of empathy. However, only 57.8% of residents openly acknowledged that the care was delayed, and 67.8% expressed a plan to prevent future errors. A few residents used rationalization (5.9%) or minimization (4.9%) behaviors; responses with these behaviors were associated with negative beta-coefficients, although this finding did not reach statistical significance. In a linear regression analysis, the strongest positive associations between resident responses and patient ratings were clustered around expressions of accountability (0.48), personal regret (0.47), apology (0.34), and intentions to prevent future mistakes (0.34).

**Conclusion:**

Resident physicians vary in which communication elements and themes they include during error disclosure, missing opportunities to meet patient expectations. While infrequent, some residents employed minimization or rationalization in their responses. Utilizing an assessment and feedback system that encourages responders to include themes layperson raters value most and to omit harmful expressions could be an important feature for future software for error disclosure communication training.

## Introduction

It is widely acknowledged that physicians should promptly disclose harmful errors to patients, explain how the error occurred, apologize, and provide patients with emotional support (1, 2). Doing so with transparency and caring fulfills ethical and regulatory obligations, minimizes patient suffering, and promotes organizational learning (3). Unfortunately, conversations after errors frequently fall short of patient expectations because they lack the information or empathy sought by patients (4, 5). Physicians report inadequate training as a key contributor to their difficulty with effective error disclosure (6–8). In response, mandates have emerged in the US for resident physicians to receive training on the disclosure of patient safety events (9), and for hospitals to support practicing physicians preparing to disclose an error (10).

To meet these requirements, educators and coaches need to instruct physicians on what to say, how to say it, and what not to say. Current guidance about how clinicians should disclose errors is informed by patient surveys, expert opinion, and limited empiric evidence that open communication and an apology can reduce patients' long-term distress (11–14). However, studies describing physicians' actual disclosure language and its relationship to disclosure efficacy are lacking (15). This gap limits the design of coaching and teaching tools that aim to narrow the difference between doctors' tendencies and the communication behaviors desired by patients.

The legal and emotional sensitivity of real-life error disclosure makes it difficult to observe or record these conversations for research. Instead, efforts to describe physician disclosure language have used surveys in which physicians are asked to select which multiple choice option is closest to how they would respond to a hypothetical error (4, 16), analysis of free text written responses to hypothetical errors (17), focus groups with care providers about their approach to communicating diagnostic delays (18), and assessments of physician performance in error disclosure simulations with standardized patients (19–22). While simulations maybe realistic for eliciting communication skills, research to date has not examined the relationship between observed communication behaviors and patients' assessments. We sought to describe this relationship through novel analysis of audio recordings of resident physician responses to a simulated medical error portrayed in the Video-based Communication Assessment (VCA). The primary purpose of this study was to describe the language residents would use to disclose an error, and to determine the association of those language choices with layperson ratings of their disclosure skills.

The VCA is a smartphone-based software tool for physicians to practice their error disclosure skills and receive feedback on their responses (23). The VCA app describes a medical case, presents a video prompt of a simulated patient, and directs the user to audio-record what they would say in response to the patient prompt in the scenario. Responses are reviewed by panels of 8–10 crowdsourced laypeople, who provide ratings on key domains of error disclosure quality on a five-point scale and qualitative responses on what would constitute the ideal response. Users receive feedback reports with a numerical summary rating and learning points derived from raters' comments (24, 25). These reports are designed so that users can self-direct learning of communication skills, and provides them with high-quality, actionable feedback, without requiring the high level of resources needed for standardized patient interactions (26). This aligns with the experiential learning framework, as it enables trainees to build their knowledge through experience and feedback (27).

In a recent multi-center randomized trial, resident physicians practiced error disclosure with the VCA and were randomized to either receive crowdsourced feedback on their performance or not. The study found that residents who received feedback had significantly higher error disclosure communication skills ratings from laypeople during their subsequent engagement with the VCA (26). Prior studies of the VCA's validity for rating physician skill (25, 26), feasibility (28), and providing actionable advice have been encouraging (23, 24). Laypeople have been shown to provide reliable and consistent VCA ratings when compared to panels of patients who have experienced harm from medical care (29). However, it is unknown whether layperson ratings of residents' communication skills align with behaviors recommended by physician faculty experts, who have traditionally assessed trainee skills in simulated patient encounters. Thus, a secondary purpose of this study was to evaluate alignment between layperson ratings and the behaviors recommended by experts.

## Materials and methods

Using an existing collection of resident audio recordings collected with the VCA, we (1) conducted a thematic analysis to identify the communication behaviors present in the responses, and (2) performed multiple linear regression to determine which of these behaviors predicted layperson quantitative ratings. Data were derived from resident physician responses to a case depicting a delayed diagnosis of breast cancer used in two studies evaluating the efficacy of the VCA for error disclosure skill development. These studies included a 2022 pre/post study (25) and a 2024 randomized trial (26) with a combined cohort of 102 Internal medicine and family medicine residents who completed the case during our period of analysis. The University of Washington IRB approved all study procedures.

### Participants, VCA case

**Figure 1:**
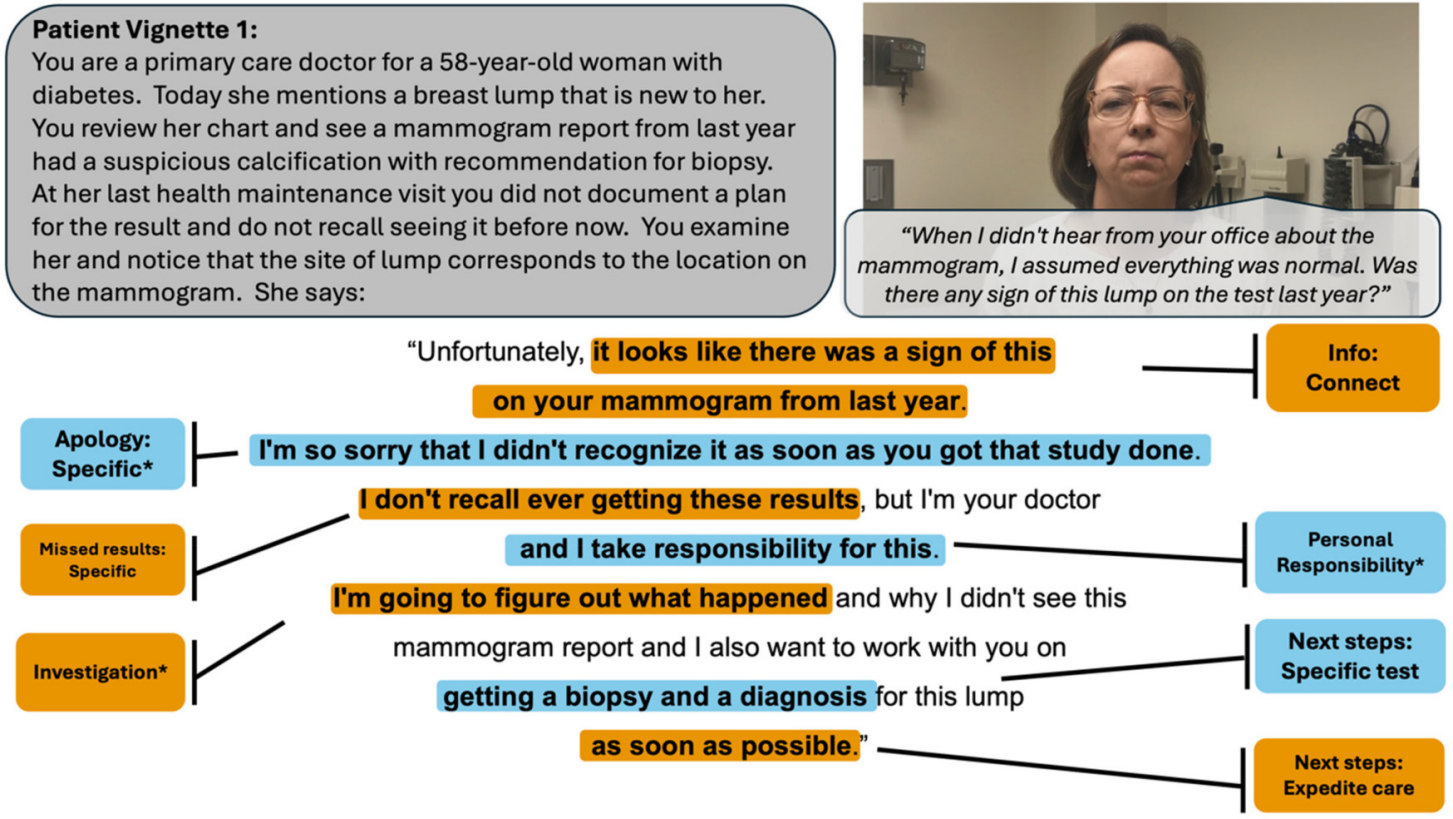
Vignette 1 prompts (displayed in gray bubble, on the left) and patient actor script (in gray bubble, on right) with an example of a highly rated resident response, annotated with the codes present. An asterisk denotes codes that belong to a thematic group significantly predictive of layperson ratings. Colors alternate for contrast only.

**Figure 2:**
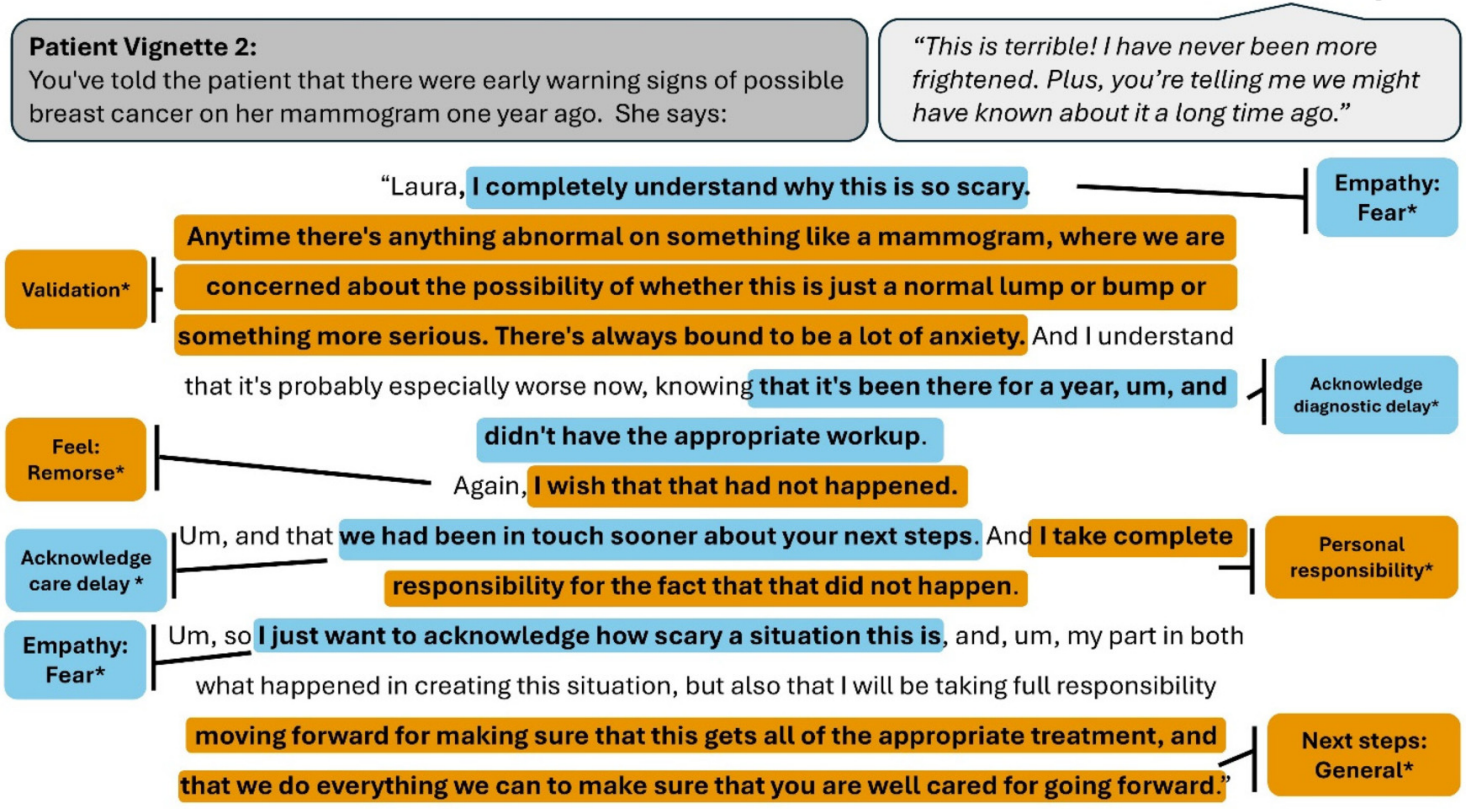
Vignette 2 prompts (displayed in gray bubble, on the left) and patient actor script (in gray bubble, on right) with an example of a highly rated resident response, annotated with the codes present. An asterisk denotes codes that belong to a thematic group significantly predictive of layperson ratings. Colors alternate for contrast only.

**Figure 3:**
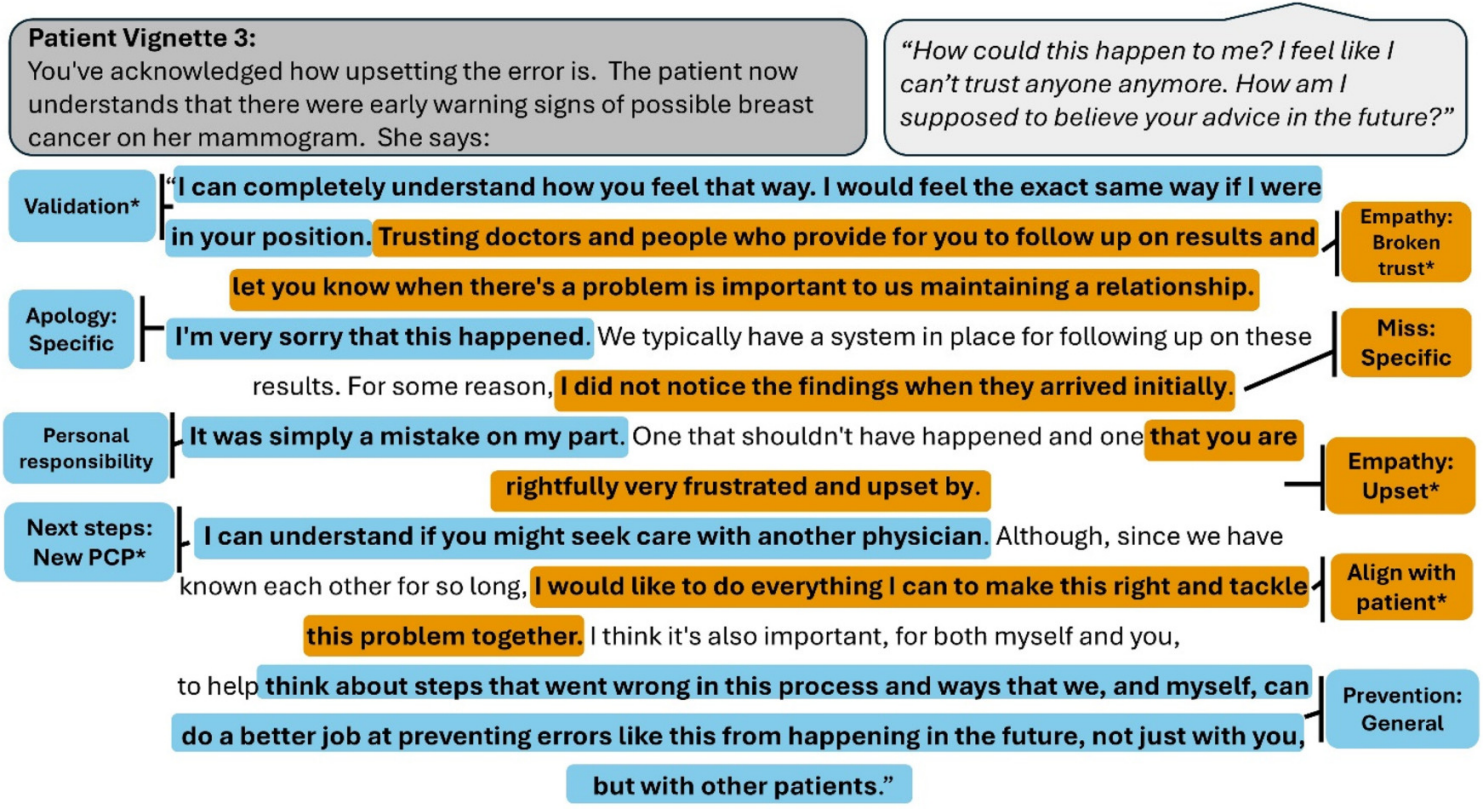
Vignette 3 prompts (displayed in gray bubble, on the left) and patient actor script (in gray bubble, on right) with an example of a highly rated resident response, annotated with the codes present. An asterisk denotes codes that belong to a thematic group significantly predictive of layperson ratings. Colors alternate for contrast only.

We recruited resident physicians from 6 Internal Medicine and Family Medicine residency programs across 5 US states to participate in classroom trials of the VCA; details of the VCA app and methods of these studies have been previously published (25, 26); essential components are summarized here. The participants were given protected time during a didactic session on error disclosure to respond to two VCA cases, including the one analyzed here. The case depicts an error in which a mammogram report concerning for breast cancer was missed by the care team for a year, leading to a delayed diagnosis. The written scenario description and the script of the video prompt are shown in the grey bubbles of Figures 1–3. The case includes three sequential segments (which will be termed vignettes) in which the actor depicts a 58-year-old patient (a) asking whether a breast lump was visible on the mammogram a year ago, (b) expressing anxious and tearful reactions to the revelation that the diagnosis was delayed by a medical error, and (c) expressing mistrust in the medical system. Residents entered the app through a personal login and password and provided audio responses to each vignette through the VCA software.

### Crowdsourced ratings

As previously described, panels of 8–10 US-based, English-speaking adults were recruited on Amazon's Mechanical Turk (MTurk) to rate physicians' recorded audio responses (25, 26). Laypeople were chosen as raters because they can be recruited rapidly and affordably, and represent the broad patient population potentially exposed to medical error. Laypeople rated each response on 6 items covering domains related to accountability, honesty, apology, empathy, caring, and overall response. Items used a 5-point scale anchored with the labels poor, fair, good, very good, and excellent. We sought at least 6 raters per response after removing raters with incomplete responses or evidence of inattention (30).

### Qualitative data analysis

We analyzed transcripts of each physician's response using a thematic approach. To develop a codebook (Supplementary Appendix 1), one co-author (EJG) first reviewed 15 responses to guide initial inductive creation of codes. Then, codes were reviewed by a set of co-authors with expertise in error disclosure communication (THG, KM, AAW) and the CANDOR error disclosure process (31). This deductive approach drew attention to the nature of the error (diagnostic delay caused by an unclear combination of physician and system factors), and the patient's primary emotional reactions manifested by the actor (fear, mistrust). We recharacterized 2 codes and added 7 codes, yielding a total of 38 codes in 15 thematic groups. Of the thematic groups, the panel recommended that error disclosure communication avoid 3 (minimization, rationalization, jargon), possibly include 2 (using patient name and personal feelings) and address the remaining 10 (acknowledge delays, acknowledge missed results, apology, accept responsibility, empathy, alignment, next steps, system improvements, financial, address mammogram results). Two co-authors (ADA and EG) reviewed and coded each transcript independently using Atlas.ti v24 (Atlas.ti Scientific Software Development GmbH; Berlin, Germany). Coding was reviewed to achieve consensus; areas of disagreement were resolved by group discussion with a third co-author (AAW).

### Quantitative data analysis

We grouped codes with similar themes for each vignette (e.g., “*acknowledge diagnostic delay”* and “*acknowledge delay of care”* were grouped together under the thematic group “*Acknowledge delays')*. We then created binary variables indicating whether a physician's response to a single vignette was coded for any code under a given thematic group. Responses with one or multiple codes within a theme were marked as “1”, whereas responses with none were marked as “0”. Binary coding was chosen to represent the presence or absence of each thematic group in a response, rather than emphasizing individual elements or their frequency.

We computed a mean overall rating score for each physician's response by aggregating lay rater's ratings across items and raters for each of the three vignettes. We chose to present aggregate scores of layperson ratings, as this scale has demonstrated high internal reliability in our prior operational research, and learners typically receive feedback in this aggregated matter. This aggregation yielded three separate communication scores for each physician (one per vignette). We then performed three separate multiple linear regressions to examine the extent to which each thematic code group predicted communication scores from lay raters for each vignette. Though the three vignettes were structured as an unfolding sequence within a broader case, we analyzed them separately to preserve time-specific contextual variation in communication behaviors that could be lost through aggregation. Certain communication behaviors, like offering an apology or acknowledging uncertainty, may have varying levels of salience depending on the phase of the encounter. Analyzing each vignette individually allowed us to capture these patterns and prevent losing granular insight that might be obscured by aggregation. Each regression model included the binary variables representing the thematic groups as predictors and the aggregated communication scores as the dependent variable. Statistical analyses were conducted using R version 4.1.

## Results

### Participant characteristics

Characteristics of the resident physician participants and layperson raters are presented in Table 1. The mean overall layperson scores for responses were 3.25 (SD = 0.49, IQR = 0.75) for vignette 1, 3.37 (SD = 0.43, IQR = 0.51) for vignette 2 and 3.51 (SD = 0.39, IQR = 0.48) for vignette 3.

**Table 1:** Resident participant and layperson rater characteristics.

Characteristic	*N*	%
(a) Resident physician characteristics (*N* = 102)		
Gender		
Female	45	44.1%
Male	52	51.0%
Non-binary	1	1.0%
Prefer not to say	4	3.9%
Specialty		
Internal Medicine	89	87.3%
Family Medicine	13	12.7%
(b) Layperson rater characteristics (*N* = 234)		
Gender		
Female	90	38.5%
Male	143	61.1%
Prefer not to say	1	0.4%
Age (years)		
18–24	10	4.3%
25–34	86	36.8%
35–44	66	28.2%
45–54	37	15.8%
55–64	28	12.0%
65+	7	3.0%
Education		
Some high school	1	0.4%
Graduated high school or equivalent	24	10.3%
Some college, no degree	29	12.4%
Associate degree	14	6.0%
Bachelor's degree	129	55.1%
Graduate degree	37	15.8%

### Communication elements present in resident responses

The prevalence of communication themes across the entire case and representative quotes are presented in the text below. Themes varied significantly across vignettes; counts and percentages per vignette are shown in Table 2.

**Table 2:** Results table showing the number and percentage of resident responses that included each code for every vignette prompt.

Group Code	Vignette 1		Vignette 2		Vignette 3	
	*N* (%) Present	*β* (SE)	*N* (%) Present	*β* (SE)	*N* (%) Present	*β* (SE)
Acknowledge Delays						
Acknowledge diagnostic delay	16 (15.7)	–	35 (34.3)	–	2 (2.0)	–
Acknowledge care delay	10 (9.8)	–	14 (13.7)	–	1 (1.0)	–
Grouping Total	26 (25.5)	0.17 (0.09)^+^	46 (45.1)	0.19 (0.08)*	3 (2.9)	−0.16 (0.22)
Acknowledge missed results						
Missed results: General	26 (25.5)	–	16 (15.7)	–	5 (4.9)	–
Missed results: Specific	45 (44.1)	–	10 (9.8)	–	2 (2.0)	–
Grouping Total	63 (61.8)	0.03 (0.08)	26 (25.5)	−0.09 (0.09)	7 (6.9)	0.03 (0.15)
Apology						
Apology: General	16 (15.7)	–	25 (24.5)	–	13 (12.8)	–
Apology: Specific	50 (49.0)	–	54 (52.9)	–	29 (28.4)	–
Grouping Total	60 (58.8)	0.34 (0.09)***	71 (69.6)	0.11 (0.09)	38 (37.3)	0.03 (0.08)
Accept responsibility						
Apology with accountability	8 (7.8)	–	8 (7.8)	–	9 (8.8)	–
Personal responsibility	25 (24.5)	–	20 (19.6)	–	13 (12.8)	–
Disclose error	8 (7.8)	–	15 (14.7)	–	37 (36.3)	–
Grouping Total	30 (29.4)	0.48 (0.09)***	32 (31.4)	0.18 (0.08)*	41 (40.2)	0.09 (0.08)
Empathy						
Empathy: Fear	3 (2.9)	–	54 (52.9)	–	8 (7.8)	–
Empathy: Upset	6 (5.9)	–	34 (33.3)	–	16 (15.7)	–
Empathy: Broken Trust	1 (1.0)	–	2 (2.0)	–	61 (59.8)	–
Validation	4 (3.9)	–	43 (42.2)	–	45 (44.1)	–
Empathy: Sad	0 (0)	–	0 (0)	–	2 (2.0)	–
Empathy: Self-blame	0 (0)	–	0 (0)	–	0 (0)	–
Empathy: Time to Process	0 (0)	–	0 (0)	–	2 (2.0)	–
Grouping Total	12 (11.8)	0.05 (0.13)	79 (77.5)	0.21 (0.1)*	89 (87.3)	0.36 (0.11)**
Physician Feelings						
Feel: personal	4 (3.9)		3 (2.9)	–	7 (6.9)	–
Feel: remorse	0 (0)		2 (2.0)	–	1 (1.0)	–
Grouping Total	4 (3.9)	0.43 (0.2)*	5 (4.9)	0.47 (0.18)*	8 (7.8)	−0.04 (0.15)
Alignment						
Align with patient	5 (4.9)		26 (25.5)	–	50 (49.0)	–
Rebuilding partnership	2 (2.0)		4 (3.9)	–	45 (44.1)	–
Grouping Total	7 (6.9)	−0.29 (0.16)^+^	29 (28.4)	0.19 (0.09)*	73 (71.6)	0.24 (0.09)**
Next steps						
Next steps: General	21 (20.6)	–	40 (39.2)	–	23 (22.6)	–
Next steps: Expedite care	3 (2.9)	–	14 (13.7)	–	8 (7.8)	–
Next steps: Specific test	10 (9.8)	–	22 (21.6)	–	15 (14.7)	–
Next steps: New PCP	0 (0)	–	1 (1.0)	–	32 (31.4)	–
Next steps: Discuss	3 (2.9)	–	5 (4.9)	–	5 (4.9)	–
Next Steps: Recommendation	1 (1.0)	–	3 (2.9)	–	1 (1.0)	–
Grouping Total	32 (31.4)	0.12 (0.09)	47 (46.1)	0.18 (0.07)*	58 (56.9)	0.17 (0.08)*
System improvement						
Prevention: General	9 (8.8)	–	14 (13.7)	–	45 (44.1)	–
Investigation	10 (9.8)	–	17 (16.7)	–	21 (20.6)	–
Error reporting	0 (0)	–	0 (0)	–	4 (3.9)	–
Prevention: specific	0 (0)	–	0 (0)	–	6 (5.9)	–
Grouping Total	14 (13.7)	0.34 (0.11)**	23 (22.6)	0.14 (0.09)	50 (49.0)	0.13 (0.08)
Financial						
Financial concerns: Acknowledge	0 (0)	–	0 (0)	–	0 (0)	–
Financial concerns: Address	0 (0)	–	0 (0)	–	1 (1.0)	–
Grouping Total	0 (0)	–	0 (0)	–	0 (0)	−0.38 (0.37)
General communication skills						
Use patient's name	44 (43.1)	0.1 (0.08)	26 (25.5)	0.03 (0.09)	25 (24.5)	0 (0.09)
General communication skills						
Use Jargon	46 (45.1)	0.02 (0.08)	6 (5.9)	−0.26 (0.18)	2 (2.0)	0.01 (0.28)
Rationalization						
Rationalize error	0 (0)	–	0 (0)	–	6 (5.9)	−0.19 (0.17)
Minimization						
Minimization	0 (0)	–	5 (4.9)	−0.24 (0.17)	0 (0)	–
Address mammogram results						
Prior results	78 (76.5)	−0.03 (0.1)	6 (5.9)	0 (0.16)	2 (2.0)	−0.35 (0.26)
Connect to current concern	58 (56.9)	0.01 (0.08)^+^	8 (7.8)	0.13 (0.15)	0 (0)	–

*β* indicates the strength and direction of the relationship between for presence of each code and layperson rating response as found by the multiple linear regression analysis.

^+^ Indicates a *p* value between 1 and.05.

* Indicates significance at *p* < .05.

** Indicates significance at *p* < .01.

*** Indicates significance at *p* < .001.

#### Explanatory clinical information

Virtually all residents (*N* = 101, 99.0%) provided some explanation of clinical information in at least one of their responses. Most (*N* = 80, 78.4%) residents referenced the findings from the prior mammogram in at least one of the three vignettes. Many (*N* = 64, 62.8%) also connected prior findings to the current concerns. For example, one resident said, “*On your mammogram last year, there was actually an indication of a suspicious calcification”* and another said “*And, that is the same area that you are now feeling this lump.”*

#### Acknowledgement that information was missed by the care team

Approximately three quarters of residents (*N* = 76, 74.5%) generally acknowledged that information was missed by the care team or provided some explanation as to why information was missed in at least one of their responses. For example, one resident said, “*I'm not sure how that was missed*”, where another said, “*I think I must have overlooked it when I was reviewing your chart prior to our last visit.*”

#### Acknowledgement of a diagnostic delay

More than half of the residents (*N* = 59, 57.8%) acknowledged a diagnostic delay or a delay in care in at least one of the three vignettes. For example, one resident stated, “*We could have known about this about a year ago,*” while another remarked, “*…your mammogram did indicate last year that there was a calcification in the area, and we should have ordered the biopsy for that.*” Very few responses (*N* = 5, 4.9%) minimized the clinical impact of the time delay. For example, one resident said, “*It may not necessarily be that this year would've made a huge difference.*”

#### An apology

Nearly all (*N* = 92, 90.2%) of residents provided responses which included either a general apology or a specific apology in at least one of the three vignettes. For example, one resident said “*I'm very sorry about this*” and another said “*I'm very sorry that I didn't tell you about this last year.*”

#### Error disclosure and personal responsibility

More than half (*N* = 66, 64.7%) of residents provided responses which included some admission that an error did occur, to at least one of the three Vignettes. For example, one resident said, “*I apologize for the mistake that happened*”, another said “*It's absolutely my fault for not following up on that and sending you for a biopsy*” and another said “*I'd like to start by saying that a mistake was made on your mammogram last year.*” Rationalization was uncommon, but a minority (*N* = 6, 5.9%) of resident responses to vignette 3 attempted to normalize the mistake. One resident said, “*although we are physicians and we do go through extensive training, we do make a mistake from time to time and after all, we are we all human*” and another said, “*As a human, it is unfortunately hard to escape making mistakes.*”

#### Empathy

Nearly all (*N* = 98, 96.1%) residents provided at least one response expressing a component of empathy. Manifestations of empathy included validating or acknowledging the patient's emotions, acknowledging that trust was broken, and offering time to process the information. For example, one resident acknowledged the patient's fears by saying “*I recognize that this can be really scary*”. Another resident acknowledged that trust was broken by saying “*it's completely understandable if you feel like you can't trust us anymore.*”

#### Personal feelings or remorse

Few (*N* = 14, 13.7%) residents provided responses which mentioned their own personal feelings to at least one of the three Vignettes. For example, one resident expressed how they were affected by saying “*I'm also very upset about this*” and another expressed remorse by saying “*I regret that this happened*.”

#### Patient alignment

Many (*N* = 77, 75.5%) residents provided at least one response expressing alignment with the patient through commitments of support, working together as a team, or an intention to regaining trust. One resident demonstrated alignment with the patient by saying “*I would like to do everything I can to help you through this, and I would like to try to support you moving forward.*” Another addressed trust by saying “*I think my hope is that over time, if we continue to work together, that I can slowly rebuild your trust in me.*” Only one resident addressed the financial aspect of the error by saying “*we can do whatever we can at this point in time financially to help resolve our error for you.*”

#### Patient-centered communication

Approximately half (*N* = 56, 54.9%) of residents used the patient's name in at least one of their responses. Regarding use of medical jargon, about half (*N* = 47, 46.1%) of residents used the term “*calcification*” without further explanation about what it meant or its clinical significance. This behavior was mostly present in responses to Vignette 1.

#### Plans for next steps in care

Many (*N* = 79, 77.5%) residents provided responses which contained mentions of next steps in care, to at least one of the three Vignettes. Responses ranged from general next steps to specific next steps, such as recommending a biopsy. Some responses made explicit recommendations for next steps or expressed that they would be expedited. Some also mentioned that they would welcome further discussion regarding the error or expressed understanding of the desire to transition to a new provider. For example, one resident said “*let's repeat your mammogram imaging now and let's get a biopsy of that area, as well*” and another said “*I will do everything in my power to get you in for biopsies as soon as possible to make sure that this is taken care of.*” One resident mentioned the possibility of switching to a new provider by saying “*Alternatively, if you don't feel comfortable following with my group anymore, I can certainly refer you to some well-respected primary care physicians in the area to establish care.*”

#### Plans to prevent future mistakes

Many (*N* = 62, 67.8%) residents provided responses which made mention of preventing future mistakes from happening again to at least one of the three Vignettes. Responses ranged from general desires to prevent mistakes to specific and concrete action items. They also mentioned plans to investigate what happened and to report to hospital systems. For example, one resident vaguely mentioned “*I will ensure that this doesn't happen again*” whereas another was more specific by saying “*Going forward, I will be sure to review every test result that I have with you personally.*” Another resident said, “*we're going to start an investigation to see what had happened.*”

### Thematic elements predictive of layperson ratings

The results of the three multiple linear regression analyses are presented in Table 2. For Vignette 1, communication themes predictive of layperson ratings on responses included Apology (*β* = .34, *p* < .001), accepting responsibility (*β* = .48, *p* = .001), physician's feelings (*β* = .43, *p* = .032), and system improvement (*β* = .34, *p* = .003) were significant predictors of layperson ratings. These elements accounted for 49% of the variance (adjusted R^2^ = .49), F (13, 88) = 8.49, *p* < .001.

For Vignette 2, communication themes predictive of layperson ratings on responses were acknowledging delays (*β* = .19, *p* < .014), accepting responsibility (*β* = .18, *p* = .038), empathy (*β* = .21, *p* = .032), physician feelings (*β* = .47, *p* = .012), alignment (*β* = .19, *p* = .028), and next steps (*β* = .18, *p* = .019) were significant predictors of layperson ratings. These elements accounted for 32% of the variance (adjusted R^2^ = .32), F (14, 87) = 4.35, *p* < .0001.

For Vignette 3, communication themes predictive of layperson ratings included empathy (*β* = .36, *p* = .002), alignment (*β* = .24, *p* = .006), and next steps (*β* = .17, *p* = .028) were significant predictors of layperson ratings. These elements accounted for 28% of the variance (adjusted R^2^ = .16), F (14, 87) = 2.42, *p* = .007.

### Concordance between expert recommendations and thematic groups predictive of layperson ratings

Regarding concordance between experts and laypeople, we found significant associations between layperson ratings and 7 of the 10 expert-recommended thematic groups. Of the expert recommended thematic groups significant predictors of layperson ratings were acknowledge delays in vignette 2 (*β* = .19, *p* < .014), apology in vignette 1 (*β* = .34, *p* < .001), accept responsibility in vignette 1 (*β* = .48, *p* = .001) and 2 (*β* = .18, *p* = .038), empathy in vignette 2 (*β* = .21, *p* = .032) and 3 (*β* = .36, *p* = .002), alignment in vignette 3 (*β* = .24, *p* = .006), next steps in vignette 2 (*β* = .18, *p* = .019) and 3 (*β* = .17, *p* = .028), and system improvements in vignette 1 (*β* = .34, *p* = .003). Though experts recommended acknowledging missed results, addressing mammogram results and addressing finances, they were not significantly predictive of layperson ratings in any vignette. Of the three thematic groups experts recommended avoiding in error disclosure (minimization, rationalization and jargon), only jargon appeared with regular frequency (45% of responses in vignette 1). However, the presence of jargon was not negatively associated with layperson ratings.

## Discussion

Our analysis of spoken responses to a hypothetical patient with a delayed diagnosis of breast cancer demonstrates significant variability in how resident physicians would approach this communication challenge. While essentially all respondents would openly address the fact that the mammogram was abnormal a year ago, residents did not reliably provide other key information, expressions of accountability, or follow-up commitments expected by patients. For example, across the whole case, only 57.8% of residents openly acknowledged that the care was delayed, and 67.8% expressed a plan to prevent future errors. These findings highlight the need to develop curricular material and practice tools that prepare each individual learner for comprehensive, open and effective error disclosure conversations. A related implication is that assessment and feedback systems should provide feedback to reinforce key behaviors that were displayed, and reminders to practice behaviors that were needed but omitted.

Our linear regression analysis provided useful insights into the behaviors that most strongly influence how laypeople rate a physician's error disclosure skills. The strongest associations were expressions of accountability (0.48), personal regret (0.47), apology (0.34), and intentions to prevent future mistakes (0.34). These behaviors varied across vignettes, corresponding to the information or response appropriate to the patient's prompt. This reflects that effective error disclosure is both an art and a science, in which the physician must both cover certain key topics, while connecting them in a clear and sensible way. The importance of these top 4 behaviors largely aligns with prior literature emphasizing the importance of accountability and apology, although the positive contribution of the physician's personal feelings is less expected (32). This could indicate that laypeople felt as though residents aligned with them through shared expression of being personally upset about the case. Although it was a relatively rare behavior (13.7% of residents), more investigation is warranted to understand how physicians can effectively express their personal feelings without taking attention away from the patient's concerns.

Several relatively uncommon communication behaviors may warrant emphasis by medical educators using the VCA or other simulations. First, only one respondent spontaneously addressed the potential financial implications of the medical error, which is a concern for many patients after medical harm. While it was likely appropriate that residents deprioritized this topic in a simulation of the early stages of disclosure, its absence suggests educators should also use simulation cases designed to elicit raising or responding to this topic as it is appropriate for providers to be prepared to respond if a patient brings up financial implications. Second, a small percentage of residents used rationalization or minimization behaviors that would likely anger or alienate patients. Responses with these behaviors were associated with negative beta-coefficients, although this finding did not reach statistical significance. Remediating this uncommon, but critical, behavior would require other approaches, such as a faculty coach (33) or using artificial intelligence to create individualized feedback (34).

Although nearly all residents offered either a general or specific apology at some point, they were spread across vignettes (e.g., 58.9% of vignette 1 responses included an apology, and 69.6% in vignette 2). Yet, apologies only appeared to influence the overall rating most when done at the outset of the conversation, suggesting that even brief delays can reduce the perceived quality of the apology. This finding warrants further investigation. Although prior literature has focused on the overall presence of an apology or its phrasing (14, 35), it may be that the timing is more relevant than previously appreciated.

The linear regression analysis identified transcript-based variables that explained up to 49% of the variance in communication performance. This is robust but suggests that factors other than word choice also contribute to how laypeople rate the error disclosure skills of physicians. This may include prosodic features such as intonation, volume, pace, and stress. Although there are ways to measure these features of speech, they fall outside the usual expertise of medical educators and may be harder to coach than language and thematic content. Yet, this suggests an opportunity to utilize both speech and language metrics to fully understand error disclosure effectiveness.

A secondary aim of this study was to examine the alignment between error disclosure content recommended by experts and the communication behaviors that contribute to higher layperson ratings of disclosure. We found that all the expert-recommended thematic groups were associated with a positive beta coefficient, and all except three (acknowledge missed results, addressing mammogram results and addressing finances) were significantly associated with higher layperson ratings**.** This adds to the validity evidence for the VCA as an assessment tool, including prior work showing that laypeople are reliable surrogates for patients who were injured by errors (29). Contrary to expert expectations, jargon was not negatively associated with layperson ratings. Despite this result, existing literature shows that jargon can hinder patient comprehension and shared decision-making (36, 37). These findings highlight the importance of integrating both patient perspectives and expert input when identifying optimal physician communication strategies.

Our study has important limitations. First, we analyzed responses to a simulated scenario, which may elicit different behaviors from real-life disclosure. For example, our results may overestimate the portion of residents who apologize or offer open explanations of the error, compared with high-stakes real scenarios. Second, the VCA does not evaluate body language or facial expressions that contribute to communication efficacy in real life, and our coding was based on transcripts alone, which excludes the contributions from tone or speaking style. Third, the VCA also does not measure encourage open-ended questions or silence, although curiosity and patience are desirable hallmarks of empathy that were not assessed in this study. Fourth, our codebook may not have captured all relevant behaviors that can be identified from text. Fifth, we did not ask layperson raters why they provided specific ratings; assessing their emotional responses and viewpoints could offer novel insight. Sixth, the sample represents residents who participated in research studies at a mix of internal medicine and family medicine residencies; findings may not generalize to other specialties and non-participants may have different communication behaviors. Lastly, this study analyzed physician-patient communication alone, and did not investigate the important role of other healthcare professions in team-based disclosure.

## Conclusion

Resident physicians vary in which components they include in error disclosure, missing opportunities to meet patient expectations. Uncommonly, some residents employed rationalization or minimization in their responses. Tailoring an assessment and feedback system to encourage responders to include the themes that layperson raters value most and to omit harmful expressions represents an important feature for future software for error disclosure communication training. Expert-recommended communication behaviors also aligned highly with the themes associated with higher ratings from laypeople, adding evidence for the validity of using VCA for error disclosure skill assessment.

## Data Availability

The original contributions presented in the study are included in the article/Supplementary Material, further inquiries can be directed to the corresponding author.
