# Supplementary material for: Error disclosure: what residents say and what patients find effective
Source: Front Health Serv. 2025 Jun 20;5:1577092. doi: 10.3389/frhs.2025.1577092 (PMC12226483; doi:10.3389/frhs.2025.1577092)
Supplement: Supplementary file 1 [file Table1.docx]

Appendix 1: Codebook by thematic group, with descriptions and examples.

| **Group** | **Code** | **Description** | **Examples** |
| --- | --- | --- | --- |
| Address mammogram results | Prior results | Provides specific information regarding the prior mammogram results | Discusses prior mammogram results (“prior abnormalities”, "suspicious area” or "calcification") |
|  | Connect to current concern | Connects the prior abnormal mammogram findings to the patient's current concerns (lump) | “…that is the same area that you are now feeling this lump” |
| Acknowledge delays | Acknowledge diagnostic delay | Acknowledges that there was a delay in sharing diagnostic information with the patient | "I think this is something we could have known about earlier" |
|  | Acknowledge care delay | Acknowledges that the delay in diagnosis resulted specifically in a delay of care | "We could have done something about it earlier" |
| Acknowledge missed results | Missed results: General | Provides some general information of the fact that the information was missed, although no details regarding what happened | "Somehow this information was missed" or "I'm not sure how this was missed" |
|  | Missed results: Specific | Provides an explanation of how and why the information may have gotten missed, and includes details regarding what happened | "I didn't see the results last year when they came back" or "they recommended following up with a biopsy, which unfortunately we did not do." |
| Apology | Apology: General | Provides a general apology to the patient | "I'm sorry" |
|  | Apology: Specific | Provides a specific apology to the patient, including what they are sorry for | "I'm sorry for…" anything that happened (including that a mistake was made) |
| Accept responsibility | Apology with accountability | Apologizes and couples this with claiming either personal or system responsibility for error | “I’m so sorry that I made this mistake” or “I have to apologize because this was an error on my part” |
|  | Personal responsibility | Claims responsibility for the mistake that was made | "This was my fault" or "I will be taking full responsibility" |
|  | Disclose error | Discloses the event as either a medical error or mistake | "This was a medical error" or "This was a mistake" |
| Empathy | Empathy: Fear | Empathizes with the patient's fear | “I recognize that this can be really scary” |
|  | Empathy: Upset | Empathizes with the patient's feelings of being upset, frustrated or angry | “I acknowledge how upsetting this error that I made for you is” |
|  | Empathy: Sad | Empathizes with the patient's feeling of sadness | “I understand that this is very depressing” |
|  | Empathy: Broken Trust | Empathizes with the patient losing trust in the provider or system | “It’s completely understandable if you feel like you can’t trust us anymore” |
|  | Validation | Validates the patient's feelings | "You have every right to feel that way" or "I would be upset too" |
|  | Empathy: Self-blame | Expressions that relieve the patient of self-blame | "This wasn't your fault" |
|  | Empathy: Time to process | Empathizes with the need for time to process information | “I want to give you the time that you need to process this information” |
| Physician feelings | Feel: personal | Comments on the provider's own personal feelings | "I feel awful about this," or has a reflection on their own personal feelings |
|  | Feel: remorse | Comments on the provider's feelings of remorse | "I wish this had not happened" or “I regret that this happened” |
| Alignment | Align with patient | Aligns with patient as a team | "I'd like to do this with you together" or "I am here with you through this" or "we'll get through this together" |
|  | Rebuilding partnership | Aligns with patient to regain trust specifically | "I'm committed to doing everything that I can to earn your trust back." |
| Next steps | Next steps: General | Discusses general next steps in treatment plan | "Let's make a plan for next steps" |
|  | Next steps: Expedite care | Discusses need for expediency in next steps of treatment | "Let's figure out what we can do to expediate the next steps in work up" |
|  | Next steps: Specific test | Discusses specific next steps in plans, including plans for a biopsy | "Let's proceed forward with the biopsy now" |
|  | Next steps: New PCP | Offers the patient's transition of care to new provider, if necessary | "I understand if you would want to get a different primary care provider" |
|  | Next steps: Discuss | Informs the patient that they are open to further discussion regarding the error | "I'm happy to talk about this more if you want" |
|  | Next steps: Recommendation | Provides a personal recommendation for next steps in care | "I think that is the most important thing in this point for proactive treatment and-" |
| System improvement | Prevention: General | Discusses general plans to prevent similar mistakes in the future | "I'm going to do everything…to make sure that … we don't make mistakes like this in the future." |
|  | Investigation | Discusses plans to investigate this error further, to provide more information for the patient | "I'm going to do everything… to make sure that this error is investigated…" |
|  | Error reporting | Discusses plans to report this error | "I will be reporting this error to our hospital's reporting system" |
|  | Prevention: Specific | Provides specific, concrete actions that will be taken to prevent similar mistakes in the future | “ I will be sure to create reminders in the chart to prevent this from happening again” |
| General communication skills | Use patient’s name | Names patient directly | "Lorna, Miss Smith, Mrs. Smith" |
|  | Use jargon | Uses medical jargon | "calcification" or "malignancy" |
| Financial | Financial concerns: acknowledge | Acknowledges financial concerns | “You may wonder who will pay for future care…” |
|  | Financial concerns: address | Provides a specific discussion of cost-mitigation or ways they will address financial aspect of error | “We can do whatever we can at this point in time financially to help resolve our error for you” |
| Rationalization | Rationalize error | Minimizes clinicians’ responsibility by saying everyone makes mistakes, or mistakes are inevitable | “As a human, it is unfortunately hard to escape making mistakes” |
| Minimization | Minimization | Minimizes the clinical impact of the delayed diagnosis | "This delay might not really matter" |
